# PW02-011 - Favorable response to anakinra in aisle patients

**DOI:** 10.1186/1546-0096-11-S1-A151

**Published:** 2013-11-08

**Authors:** H Ozdogan, M Gattorno, S Ugurlu, M Di Rocco, G Hatemi, D Ustek, A Gul

**Affiliations:** 1 Cerrahpasa Faculty of Medicine, Istanbul University, Istanbul, Turkey; 2 EULAR Centre of Excellence in Rheumatology 2008-13, "G. Gaslini" Scientific Institute, Genova, Italy; 3 Institute for Experimental Medicine, Istanbul University, Istanbul, Turkey; 4 Istanbul Faculty of Medicine, Istanbul University, Istanbul, Turkey

## Introduction

We previously described a new autosomal recessively inherited autoinflammatory syndrome in two Turkish patients, who were second-degree cousins. Their clinical features included recurrent inflammatory attacks lasting 3-10 days since the first year of life, which are characterized by fever, erythematous or urticarial rash with hyperesthesia, serositis and edema on the face and extremities. Both patients eventually developed a lymphedema symmetrically affecting lower extremities and genitalia. Using a homozygosity mapping approach and targeted capture array based sequencing, we identified a homozygous insertion mutation in the exon 3 of the MyoD family inhibitor domain containing gene (MDFIC), causing a frameshift and inhibiting the translation of its functional cysteine-rich C-terminal domain. We herein report another patient of Italian origin and also their response to anakinra treatment.

## Case report

Italian patient, a 11 year-old girl healthy unrelated parents, had a history of caesarean section at 33th week of pregnancy, because of fetal subcutaneous tissue edema and pleural effusion. Within the first 24 h, she developed respiratory distress, and pleural effusion was drained the intensive care unit. She required ventilatory support, antibiotics and diuretics for 2 months. At the age of 9 months hearing loss due to mutation 35delG of GJB2 gene was detected and cochlear implantation was subsequently done. At the age of 2 yr, she started to have attacks of fever, limb edema, severe pleural and pericardial effusions requiring urgent pleural and pericardial fluid drainage. Laboratory investigations showed a severe anemia and elevated acute phase response, and she only responded to high dose methylprednisolone. At the age of 8 yr, she had recurrent attacks during tapering of steroids. Anakinra was started at the dosage of 2 mg/kg/day with a dramatic control of inflammatory manifestations and acute phase response. After 6 months of continuous treatment anakinra was tapered to every other day injections. Sequencing of the MDFIC gene of this patient revealed the same insertion mutation causing a frameshift.

Similarly, two Turkish cousins first tried canakinumab 150 mg every 4 weeks, but they continued to experience attacks on this treatment up to 3 to 4 months. Their treatment switched to anakinra during the last 4 months, and a favorable response (complete in the elder, and partial in the younger) was observed in both clinical and laboratory findings.

## Discussion

Identification of the same MDFIC gene mutation in an Italian patient with similar manifestations confirms the association of this variation with AISLE. Differential response to anakinra in those patients may suggest the importance of IL-1 alpha in its pathogenesis.

## Disclosure of interest

None declared

